# Supplementary figures and images for: Shifts in gut and vaginal microbiomes are associated with cancer recurrence time in women with ovarian cancer
Source: PeerJ. 2021 Jun 17;9:e11574. doi: 10.7717/peerj.11574 (PMC8214851; doi:10.7717/peerj.11574)

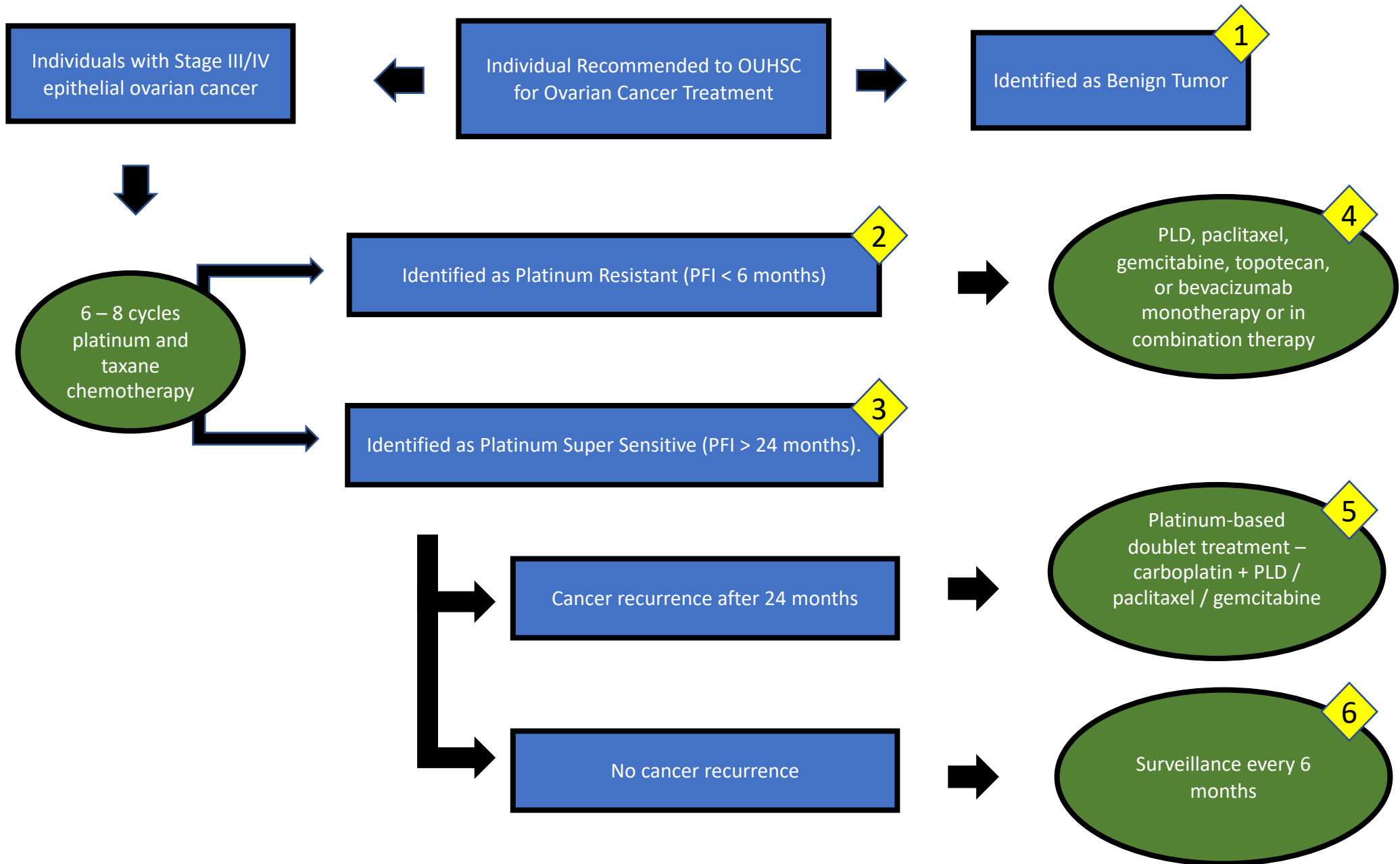

Supplement: Supplemental Information 1 — Flowchart of sample collection for individuals in different study categories. Green circles represent treatment and yellow diamonds represent periods of sample collection. Individuals with benign tumors (n = 5) had samples collected immediately (1) and received no further treatment. Those with Stage III/IV epithelial cancer went through 6-8 cycles of platinum chemotherapy. Individuals enrolled in the study had samples collected as soon as they were identified as PFI < 6 months (2) or PFI > 24 months (3). Individuals were also enrolled in the study if they had previously been identified as PFI < 6 months or PFI > 24 months (prior to the start of the study) and samples were collected during treatment (Baldwin et al., 2012; Raja, Chopra & Ledermann, 2012) or during surveillance (Dasari & Tchounwou, 2014). [file peerj-09-11574-s001.pdf]

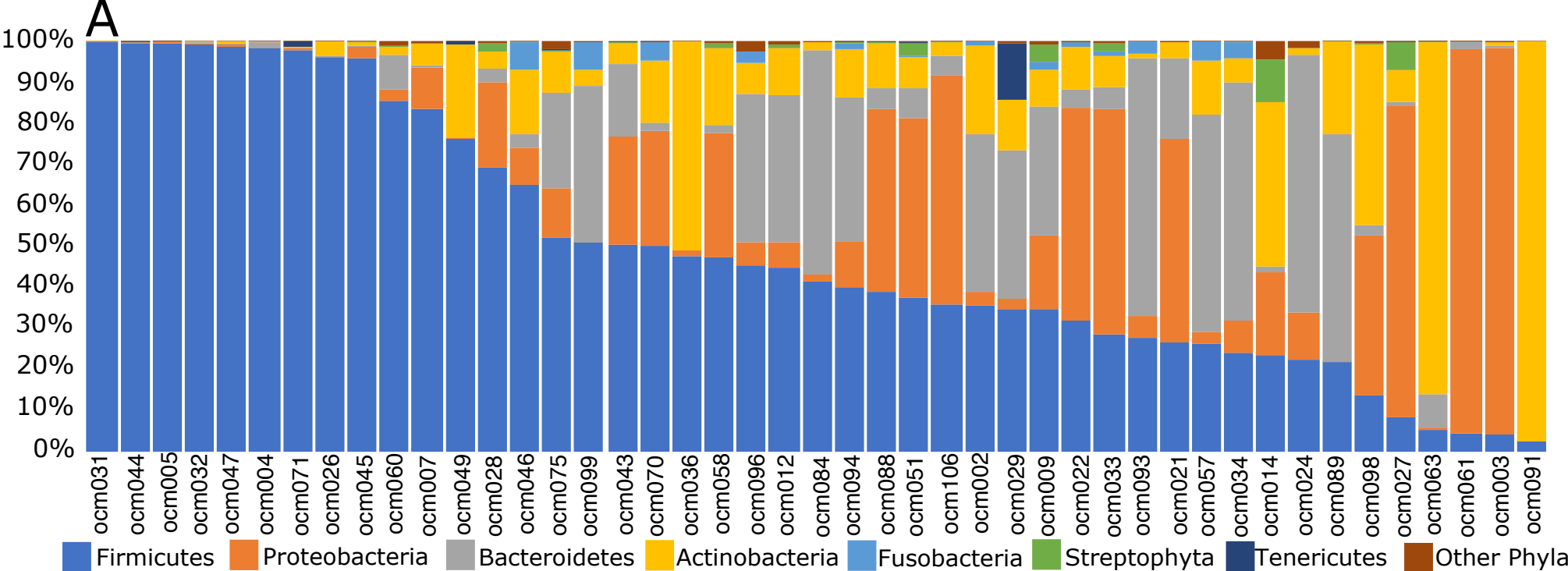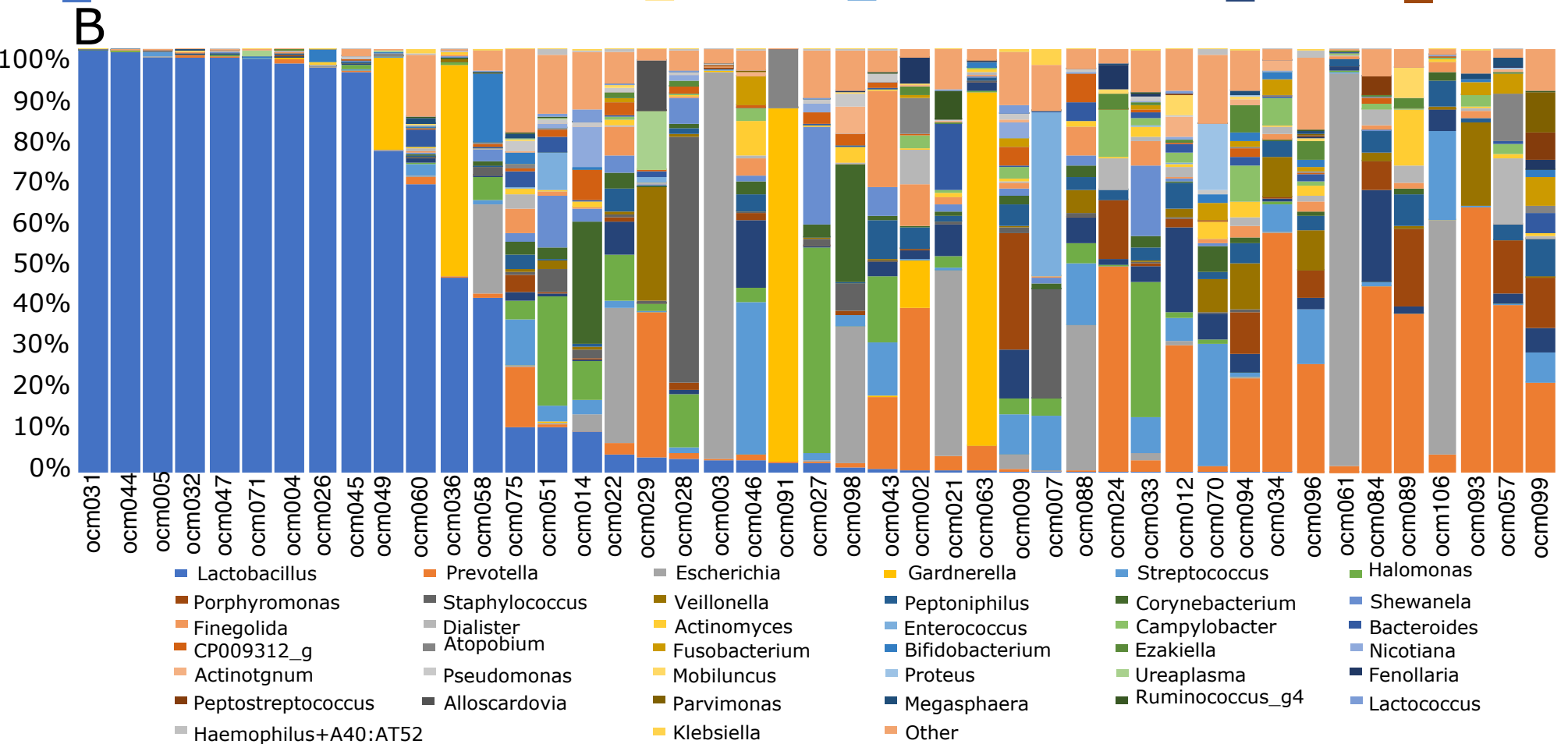

Supplement: Supplemental Information 3 — Stacked bar chart shows relative contribution of each phylum (A) and genus (B) to each individual’s vaginal microbiome and contributions from low abundance phyla and genera were combined into ‘Other’. Samples are organized along the x-axis by their relative abundance of the most dominant bacterium (Firmicutes/Lactobacillus). There was no clustering in producing this figure. Overall, the vaginal microbiome is dominated by the common vaginal bacteria: Firmicutes, Proteobacteria, and Bacteroidetes at the phylum level, and Lactobacillus, Prevotella, Escherichia, Gardnerella, and Streptococcus at the genus level. [file peerj-09-11574-s003.pdf]

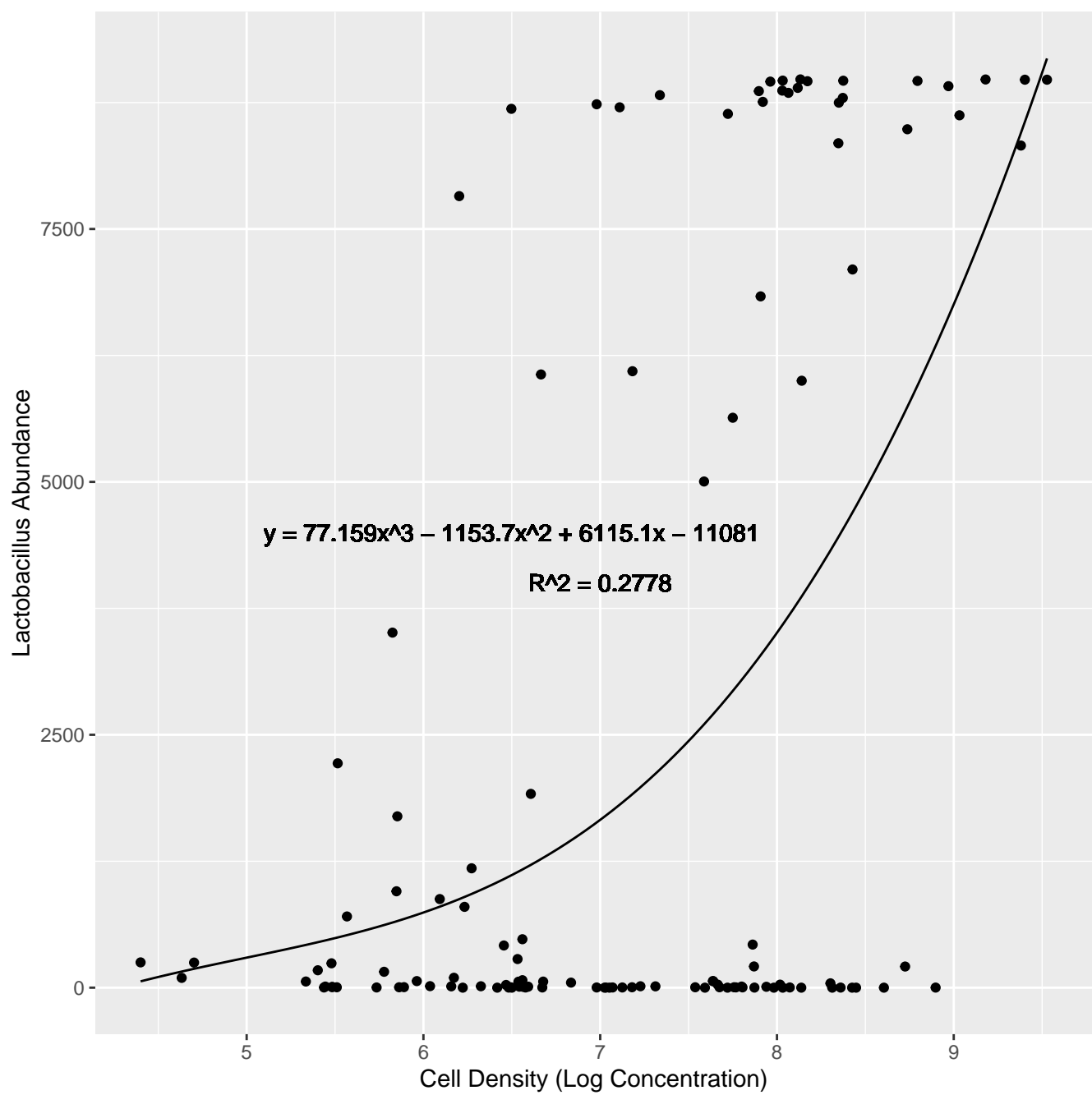

Supplement: Supplemental Information 4 — Cubic polynomial fit to log cell density (x-axis) and reads mapping to Lactobacillus (y-axis). Log cell density is calculated from a qPCR standard curve created from standards with known concentration. The positive relationship (R2 = 0.2778) indicates that Lactobacillus abundance is related to microbial concentration in the vaginal environment. [file peerj-09-11574-s004.pdf]

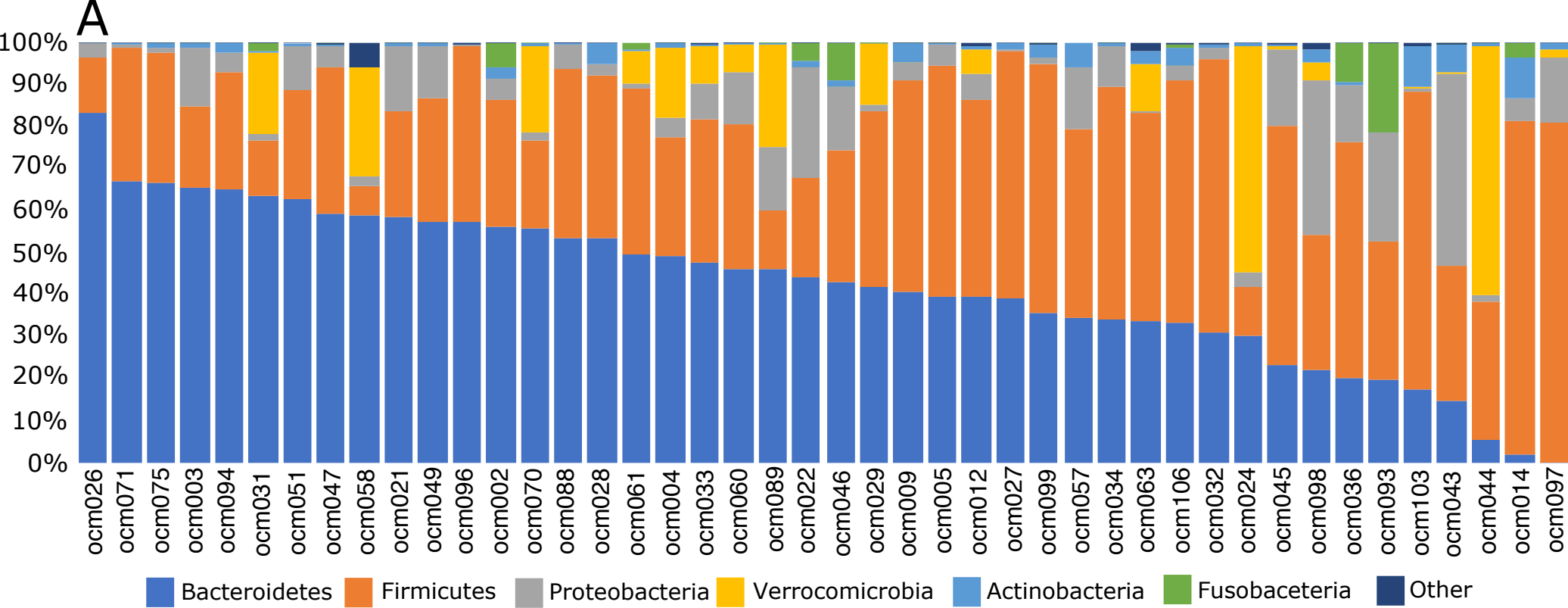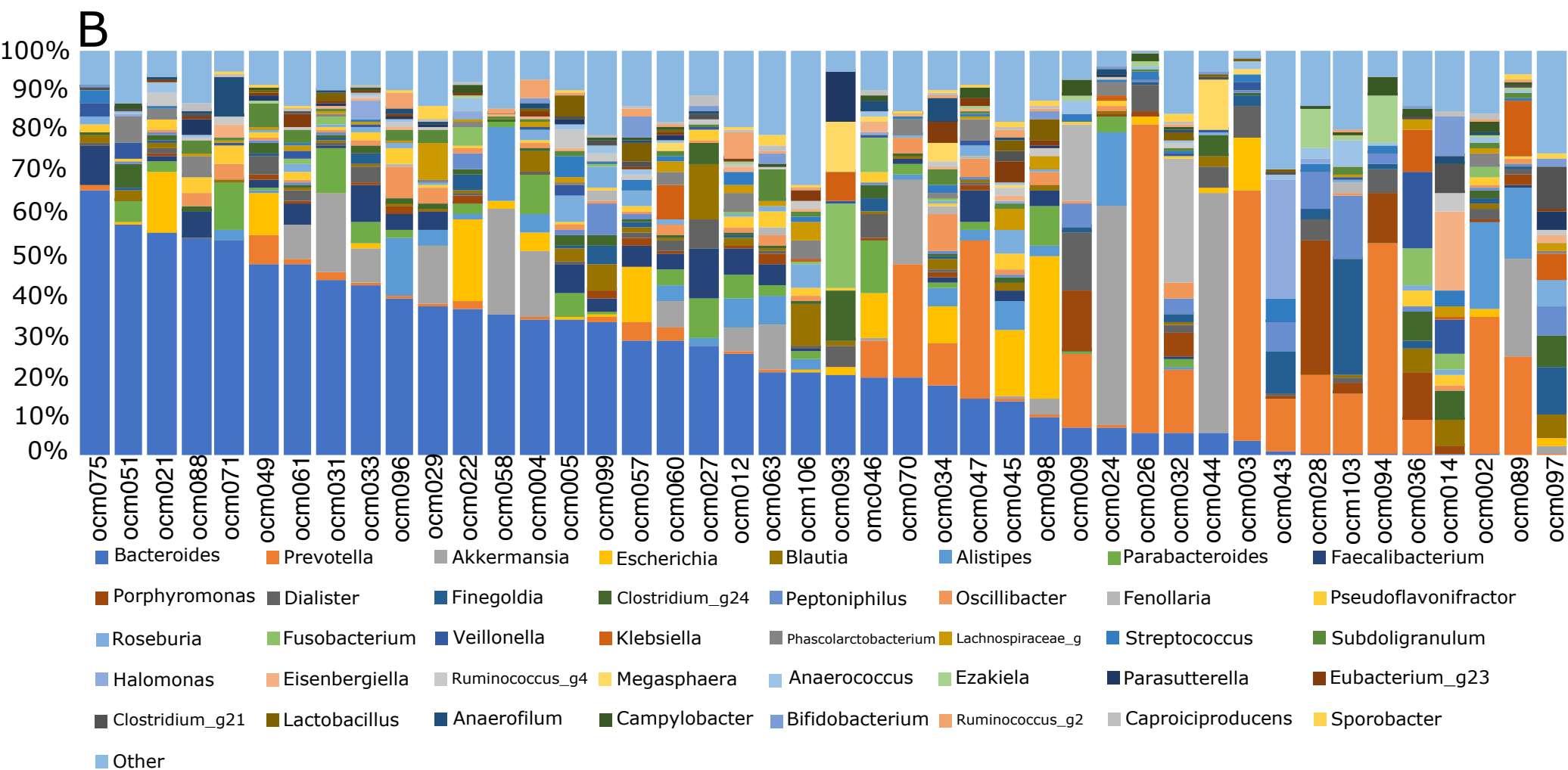

Supplement: Supplemental Information 5 — Stacked bar chart shows relative contribution of each phylum (A) and genus (B) to each individual’s gut microbiome and contributions from low abundance phyla and genera were combined into ‘Other’. Samples are organized along the x-axis by their relative abundance of the most dominant bacterium (Bacteroidetes/Bacteroides). There was no clustering in producing this figure. Overall, the gut microbiome is dominated by the common gut phyla/genera: Bacteroidetes, Firmicutes, and Proteobacteria, at the phylum level, and Bacteroides, Prevotella, and Akkermansia at the genus level. [file peerj-09-11574-s005.pdf]

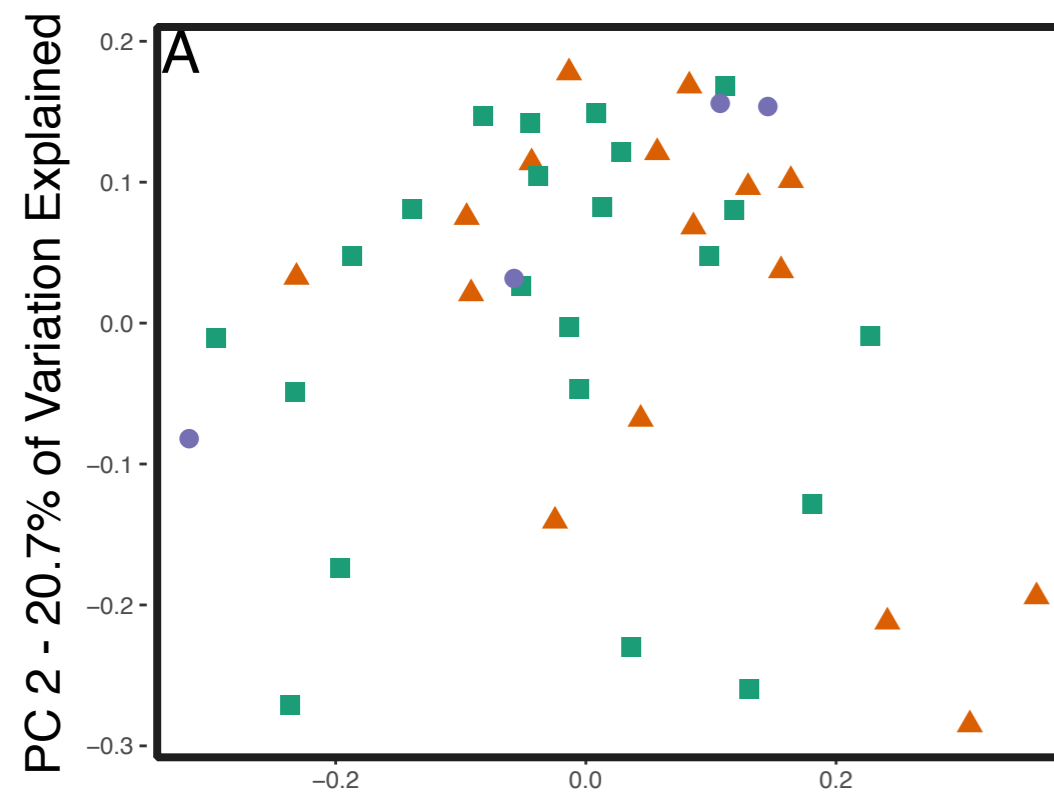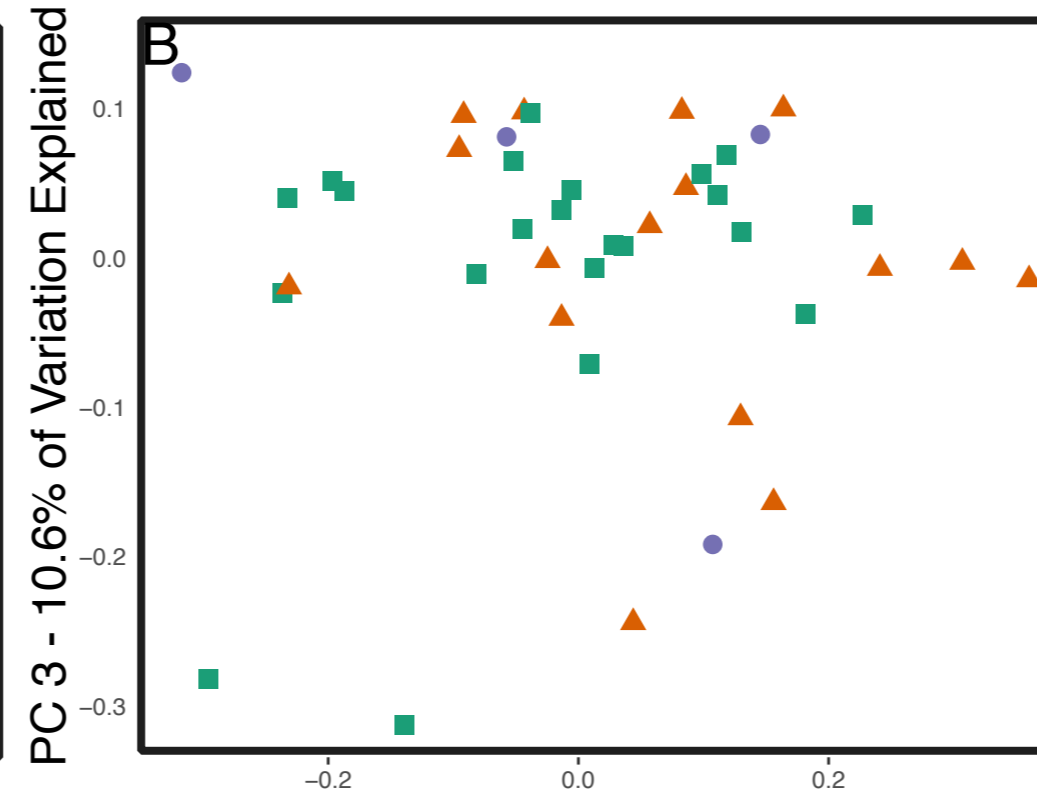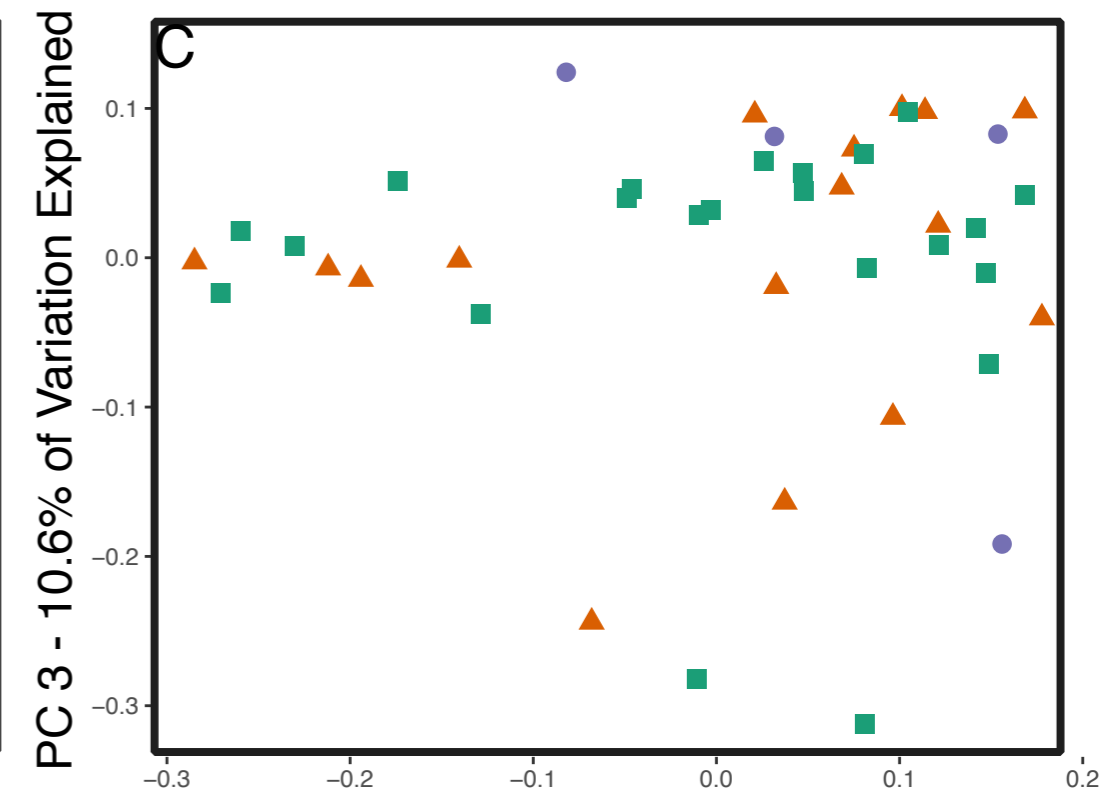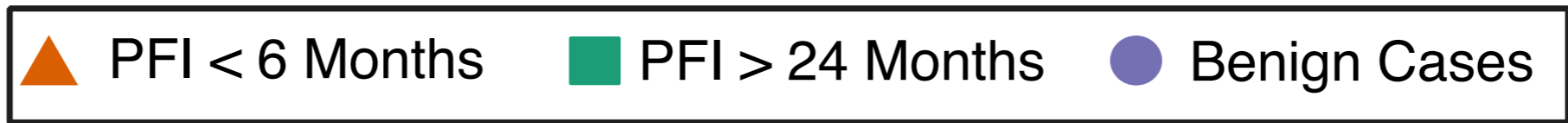

Supplement: Supplemental Information 6 [file peerj-09-11574-s006.pdf]

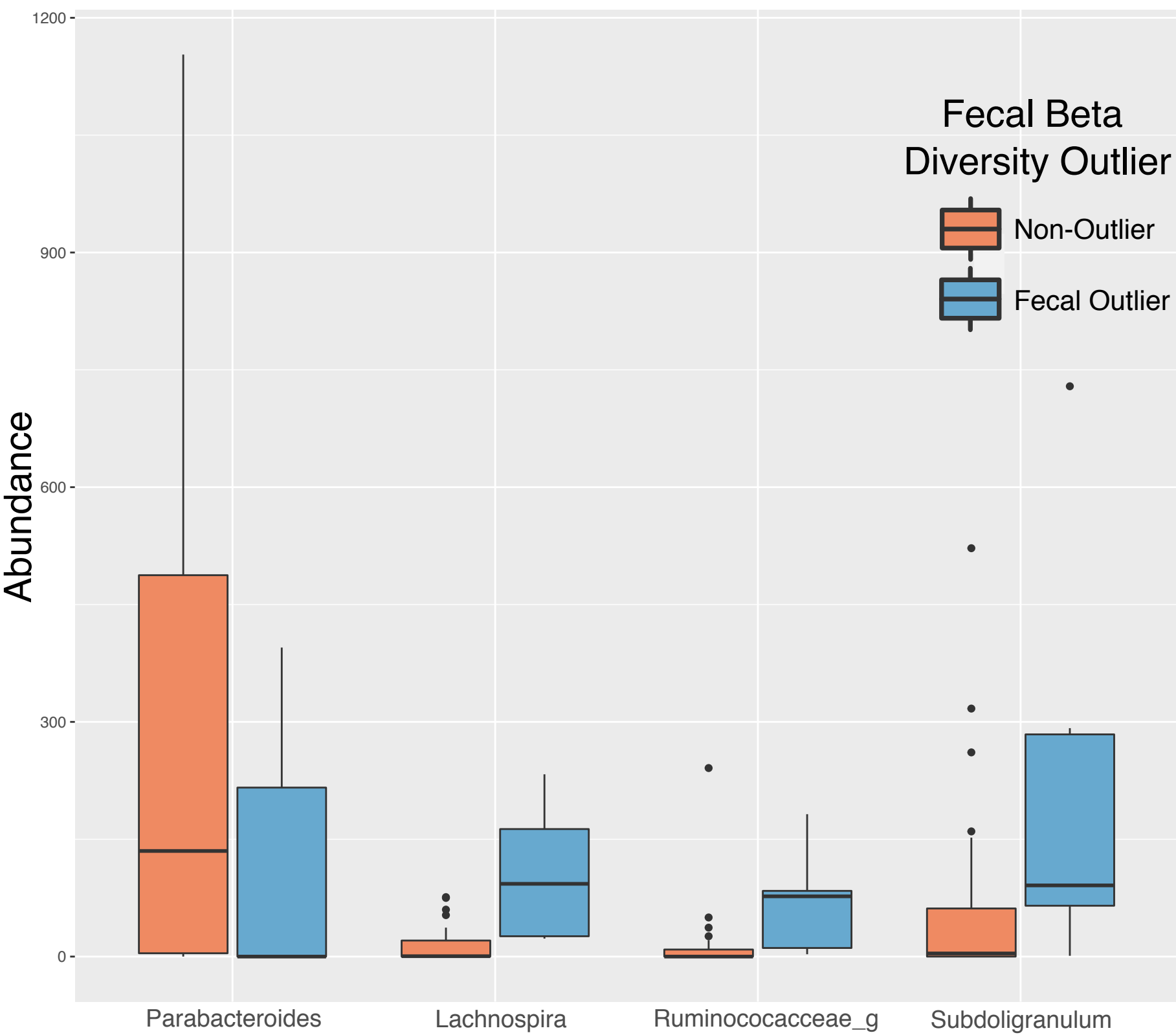

Supplement: Supplemental Information 7 — Genera belonging to the Clostridiales order (Lachnospira, Ruminococacceae, Subdoligranulum) are at higher abundance in the gut microbiome beta diversity outlier group (Krukal-Wallis p-values = 0.00039, 0.001337, 0.01121). Parabacteroides is not significantly more abundant in the non-outlier group (Krukal-Wallis p-value = 0.1031). [file peerj-09-11574-s007.pdf]

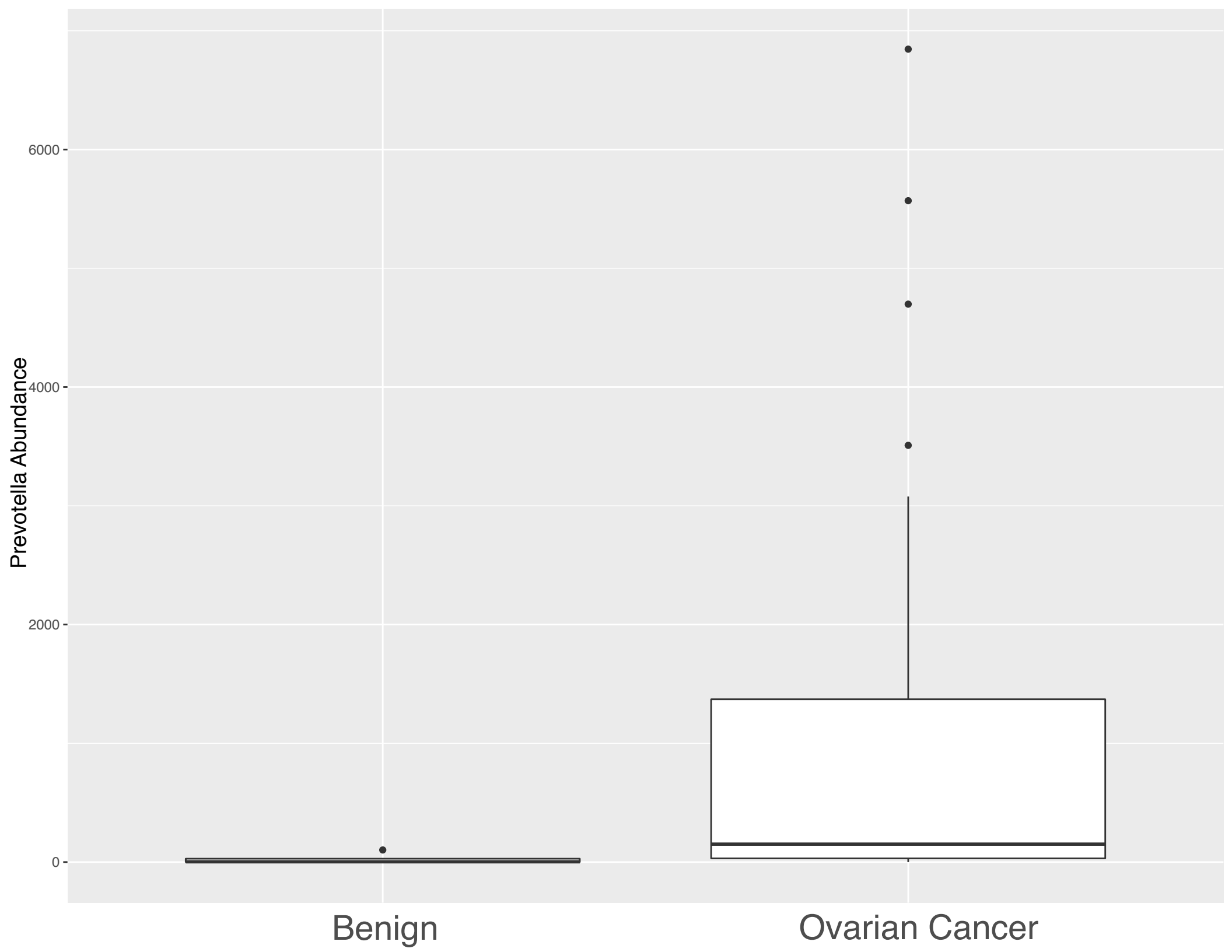

Supplement: Supplemental Information 8 — Patients with ovarian cancer (both platinum-sensitive and platinum-resistant) have higher levels of Prevotella in the gut microbiome compared to controls (p = 0.028). [file peerj-09-11574-s008.pdf]

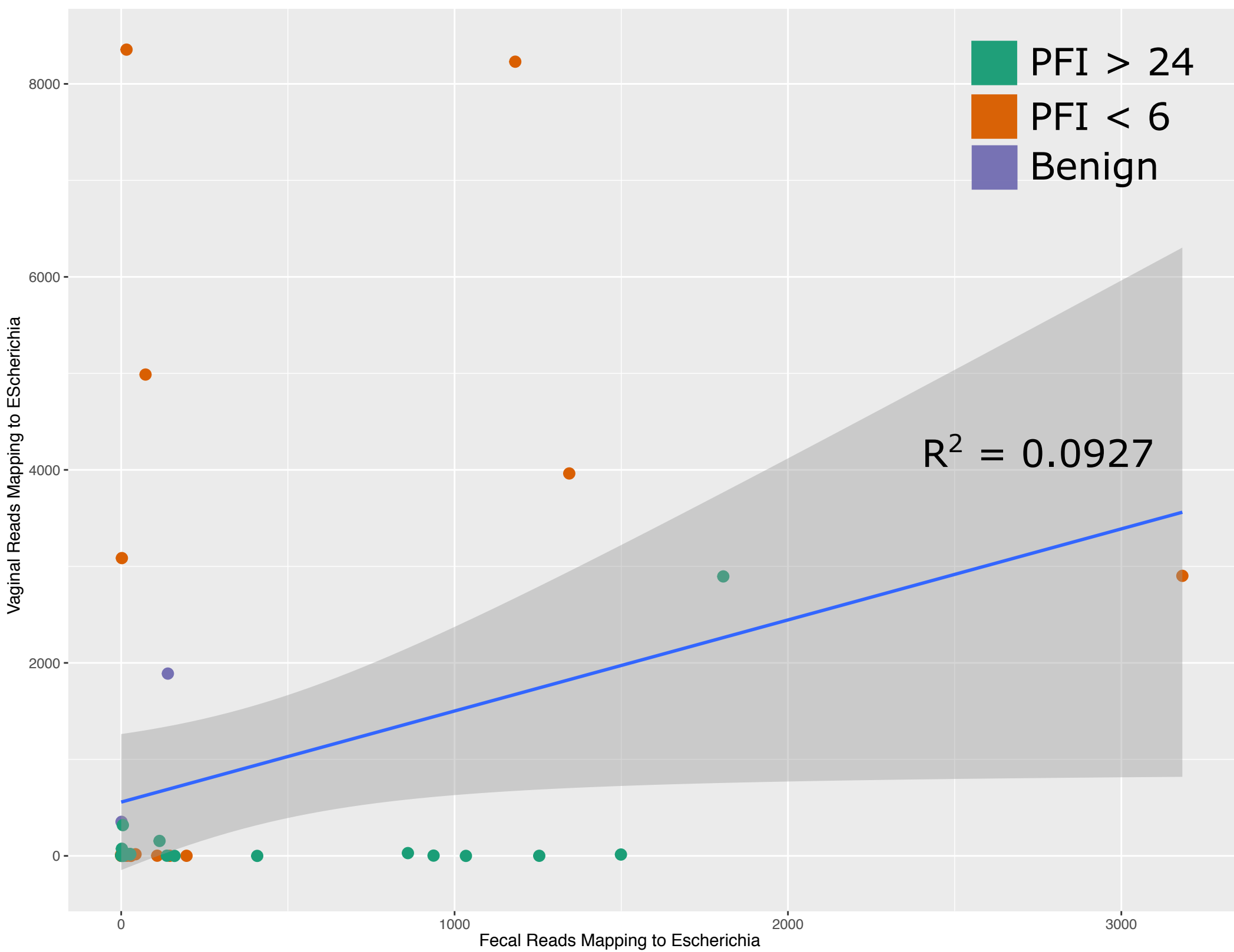

Supplement: Supplemental Information 9 — Abundance of Escherichia in the gut microbiome (x-axis) and the vaginal microbiome (y-axis). There is a very weak positive relationship (R2 = 0.09) between Escherichia abundance in the gut and vaginal microbiome. This indicates vaginal Escherichia abundance is not due to fecal Escherichia abundance. [file peerj-09-11574-s009.pdf]
