# Supplementary material for: Shifts in gut and vaginal microbiomes are associated with cancer recurrence time in women with ovarian cancer
Source: PeerJ. 2021 Jun 17;9:e11574. doi: 10.7717/peerj.11574 (PMC8214851; doi:10.7717/peerj.11574)
Supplement: Supplemental Information 2 — Each colored label is a sample from that patient number and colored lines point to the exact location for that label. Black lines connect samples from the same individual. Samples originating from the same individual had similar taxonomic composition. This informed our decision to combine data from the three vaginal samples per individual into a single sample per individual. Numbers within boxes represent sample ID. [file peerj-09-11574-s002.pdf]

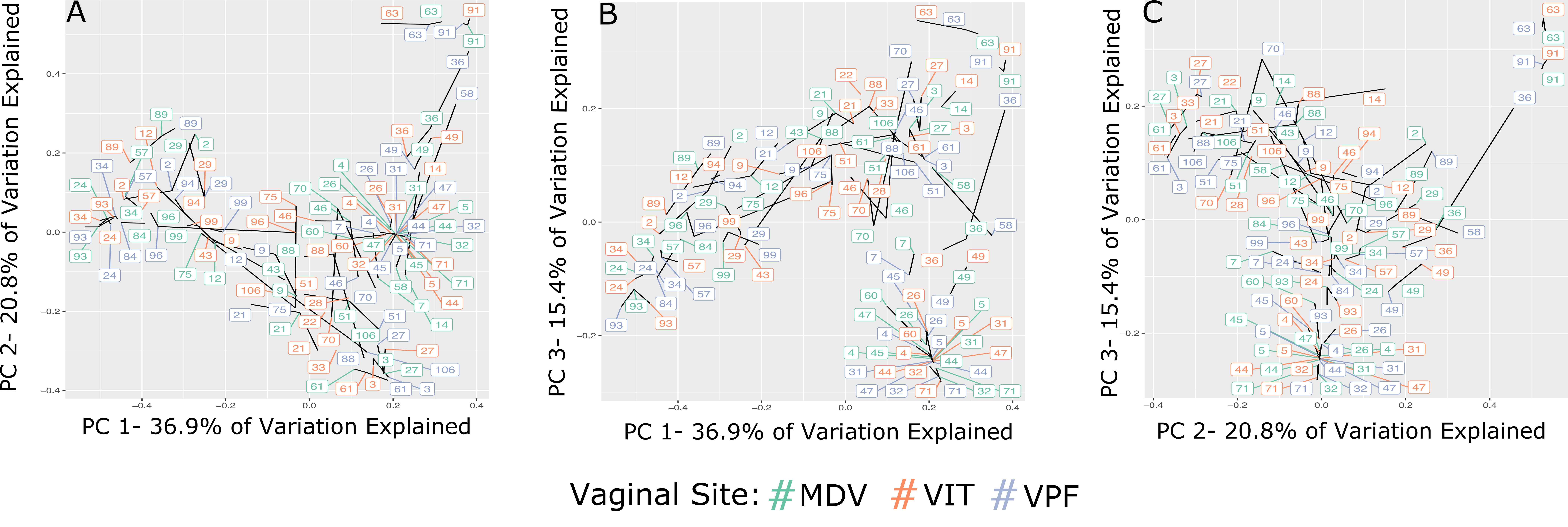
A
B
C
PC 1- 36.9% of Variation Explained
PC 2- 20.8% of Variation Explained
PC 3- 15.4% of Variation Explained
PC 1- 36.9% of Variation Explained
PC 3- 15.4% of Variation Explained
PC 2- 20.8% of Variation Explained
Vaginal Site: # MDV # VIT # VPF
