# Supplementary material for: Shifts in gut and vaginal microbiomes are associated with cancer recurrence time in women with ovarian cancer
Source: PeerJ. 2021 Jun 17;9:e11574. doi: 10.7717/peerj.11574 (PMC8214851; doi:10.7717/peerj.11574)
Supplement: Supplemental Information 14 [file peerj-09-11574-s014.docx]

**Supplementary Information**

*Failed Sequencing*

11 of the 45 individuals (24% of individuals) had failed sequencing for at least one vaginal specimen. Failed sequencing was indicated by less than 9000 reads per sample. The 11 individuals with at least one failed vaginal site were: OCM007, OCM014, OCM022, OCM028, OCM033, OCM043, OCM045, OCM058, OCM060, OCM084, and OCM094. Three individuals (OCM022, OCM028, and OCM033) had only one vaginal site successfully sequenced. VIT failed to sequence in five individuals, MDV failed to sequence in four individuals, VPF failed to sequence in six individuals. Seven of the eleven individuals had a diverse community, two had *Lactobacillus*, one had *Prevotella*, and one had *Escherichia*. We chose to pool all vaginal sites from each individual into a single site for analysis due to the ambiguity introduced when analyzing different a composition of individuals if each vaginal site was analyzed separately.

*Escherichia origin*

Although *Escherichia* is commonly found in the vaginal microbiome, it has not been reported at such high levels (>50% relative abundance) before. Because *Escherichia* is a common lab-grown bacterium and is often found in the gut microbiome (80-82) we further investigated whether the *Escherichia* abundance in our samples was due to sample contamination. We did not find evidence to support *Escherichia* contamination as the source of *Escherichia* observed in this study. First, our extraction and PCR blanks sequenced in this study report a low number of total reads and the percent of extraction/PCR blank sequencing reads mapping to *Escherichia* compared to the average read depth in this study is minimal (median = 0.0052%, range 0 – 3%, Table S2). The very low number of *Escherichia* reads in our negatives and blanks indicates any contribution of lab/environmental *Escherichia* to the high relative abundance in our vaginal samples is miniscule. Second, we observe a weak relationship between abundance of *Escherichia*  in the gut and vaginal microbiomes (R^2^ = 0.0927, Fig. S9). If fecal contamination of vaginal swabs was an issue, we would expect high fecal *Escherichia* abundance to co-occur with high vaginal *Escherichia* abundance, but this is not the case.

*Code*

Below are the steps used to process the 16S rRNA reads. Starting with non-demultiplexed fastqs

### Adapter removal for each set of samples forward and reverse

AdapterRemoval --file1 allReads_R1.fastq.gz --file2 allReads_R2.fastq.gz --trimns --trimqualities --minquality 30 --minalignmentlength 40 --maxns 0 --collapse --gzip --basename allReads

### Determine sequence header names for reads that merged

grep "^@M" allReads.merge.fastq | awk '{print$1}' | sed 's/^@M_//' > seqnames.final

### in qiime, use sequence names to pull out the associated barcodes with each read

filter_fasta.py -f index_file_I1.fastq -s seqnames.final -o filtered_barcodes.fastq

### demultiplex merged reads. Requires a properly formatted qiime metadata file

split_libraries_fastq.py -i allReads.merge.fastq -m metadata.txt -b filtered_barcodes.fastq -o demultiplexed_directory

###At this step we went back and combined reads different vaginal from same individual into a single sample.

### with seqs.fna from demultiplexed directory, use custom script to count how many times each sequence occurs

perl Make_unique.pl seqs.fna > derep.fna

### Eliminate sequences that occur less than 5 times.

usearch -sortbysize derep.fna -fastaout filterabundance.fa -minsize 5

### Cluster the filtered sequences by sequence similarity to create OTUs at 97%. This creates the de novo database

usearch -cluster_otus filterabundance.fa -otus rep_db.fa -relabel Parse

### Cluster the demultiplexed sequences against the newly created database

usearch -usearch_global seqs.fna -db rep_db.fa -strand plus -id 0.97 -uc otu_table.uc

### convert output of clustering to a otu table in tab separated format, custom script

perl Convert_uc_otu_map.pl otu_table.uc otu_table.txt

## assign taxonomy command using eztaxon datbase

assign_taxonomy.py -i otus.fa -r eztaxon.fasta -t /home/eztaxon.txt -o assigned_taxa

### make the otu table with taxonomies

make_otu_table.py -i otu_table.txt -t assigned_taxa/rep_db_tax_assignments.txt -o otus.biom

### Rarify your biom file, typically to 10,000 reads. Repeat with smaller steps to get rarefaction curve

parallel_multiple_rarefactions.py -i otus.biom -x 100 -m 10 -s 10 -n 10 -o rarefactions -O 16

### create tree that will be used for phylogenetic analysis. Two step command

mafft rep_db.fa > file_for_tree.fa

make_phylogeny.py -i file_for_tree.fa -o rep_tree.tre

### Generate alpha diversity metrics for rarefied sequences

parallel_alpha_diversity.py -i rarefactions -m observed_otus,PD_whole_tree -t rep_tree.tre -o alpha_diversity -O 8

### Collate information from alpha diversity output into one file

collate_alpha.py -i alpha_diversity -o collate_alpha

### Make rarefaction plots with alpha diversity metrics

make_rarefaction_plots.py -i collate_alpha -m metadata.txt -o rareplots

### Generate beta diversity metrics (weighted and unweighted UniFrac distances) that will be used to create PCoA plots. The depth of sequences used depends on the rarefaction plots.

beta_diversity.py -i rarefactions/rarefaction_10000_0.biom -t rep_tree.tre -o beta_diversity

principal_coordinates.py -i weighted_unifrac_rarefaction_10000_0.txt -o weighted.pc

principal_coordinates.py -i unweighted_unifrac_rarefaction_10000_0.txt -o unweighted.pc

### Generate a summary of taxa abundance for each sample

summarize_taxa.py -i rarefaction_9000_0.biom -a -L 1,2,3,5,6,7 -o taxa_summarized

### The L2, L6, and L7 files in the taxa_summarized folder were used for downstream statistical analysis.
